# Characterization of Alzheimer’s Disease-Associated Excitatory Neurons *via* Single-Cell RNA Sequencing Analysis

**DOI:** 10.3389/fnagi.2021.742176

**Published:** 2021-11-08

**Authors:** Fanghong Shao, Meiting Wang, Qi Guo, Bowen Zhang, Xiangting Wang

**Affiliations:** ^1^Department of Geriatrics, Gerontology Institute of Anhui Province, The First Affiliated Hospital, Division of Life Sciences and Medicine, University of Science and Technology of China, Hefei, China; ^2^Anhui Provincial Key Laboratory of Tumor Immunotherapy and Nutrition Therapy, Hefei, China; ^3^Division of Life Sciences and Medicine, School of Life Sciences, University of Science and Technology of China, Hefei, China; ^4^College of Liren, Yanshan University, Qinhuangdao, China; ^5^Department of Neurobiology and Biophysics, Division of Life Sciences and Medicine, University of Science and Technology of China, Hefei, China

**Keywords:** Alzheimer’s disease, single-cell sequencing, 5XFAD mice, excitatory neurons, netrin G1

## Abstract

The detailed characteristics of neuronal cell populations in Alzheimer’s disease (AD) using single-cell RNA sequencing have not been fully elucidated. To explore the characterization of neuronal cell populations in AD, this study utilized the publicly available single-nucleus RNA-sequencing datasets in the transgenic model of 5X familial Alzheimer’s disease (5XFAD) and wild-type mice to reveal an AD-associated excitatory neuron population (C3:Ex.Neuron). The relative abundance of C3:Ex.Neuron increased at 1.5 months and peaked at 4.7 months in AD mice. Functional pathways analyses showed that the pathways positively related to neurodegenerative disease progression were downregulated in the C3:Ex.Neuron at 1.5 months in AD mice. Based on the differentially expressed genes among the C3:Ex.Neuron, four subtypes (C3.1–4) were identified, which exhibited distinct abundance regulatory patterns during the development of AD. Among these subtypes, the C3.1 neurons [marked by netrin G1 (Ntng1)] exhibited a similar regulatory pattern as the C3:Ex.Neuron in abundance during the development of AD. In addition, our gene set variation analysis (GSEA) showed that the C3.1 neurons, instead of other subtypes of the C3:Ex.Neuron, possessed downregulated AD pathways at an early stage (1.5 months) of AD mice. Collectively, our results identified a previously unidentified subset of excitatory neurons and provide a potential application of these neurons to modulate the disease susceptibility.

## Introduction

Alzheimer’s disease (AD) is a neurodegenerative disease, characterized by progressive cognitive decline, and first described by Alois Alzheimer in the early twentieth century (Ballard et al., 2011; Bondi et al., 2017). The main pathological features of AD include neuronal death and synaptic degeneration (Lyketsos et al., 2011). Although neurons have developed mechanisms to protect themselves against inflammatory attacks and neurological disorders, some neurons are defective in AD. The intracellular neurofibrillary tangle formed by hyperphosphorylated tau protein and the deposition of extracellular amyloid plaques in these defective neurons are the pathological hallmarks of AD (Gao et al., 2018; Zhang and Zheng, 2019). 5X familial Alzheimer’s disease (5XFAD) model mice with mutated amyloid beta *precursor protein* (*APP*) and *presenilin* (*PSEN1* and *PSEN2*) genes are widely used in AD studies, as they quickly simulate the main pathological features of amyloid protein (Oakley et al., 2006).

A complete understanding of AD cellular functions is challenging owing to its heterogeneity. The presence of non-neuronal cells (microglia and astrocytes) in 5XFAD mice was recently attributed to the onset and escalation of AD and aging, using single-cell sequencing (Keren-Shaul et al., 2017; Habib et al., 2020). Similar results were obtained from human-derived microglia using single-cell analysis (Olah et al., 2020). Myelin formation is suggested to play a role in the pathophysiology of AD through the construction of neuronal cell atlas *via* single-cell sequencing (Mathys et al., 2019). Analysis of the olfactory bulb single-cell atlas in patients with AD revealed that a set of transcriptional factors (TFs) from specific cell populations is the driving factor that mediates the transition of AD (Grubman et al., 2019). Despite these advances, the detailed characteristics of neuronal cell populations during AD development are yet to be fully elucidated.

In the present study, the publicly available single-nucleus RNA-sequencing data of wild-type (WT) and AD mouse hippocampus at seven developmental time points (Supplementary Table 1; Habib et al., 2020) was used to cluster the cells into 13 populations. Excitatory neurons (C3:Ex.Neuron) and astrocytes (C10:Astrocyte2) were significantly altered between AD and WT mice at 1.5, 4, 4.7, 7, and 13 months. The changes in C10:Astrocyte2 abundance and its enriched functional pathways in immune-related terms were consistent with previous findings (Habib et al., 2020), suggesting the accuracy of the present study. Further analysis revealed that C3:Ex.Neuron levels increased at the early stage (1.5 months) of AD. The features of C3:Ex.Neuron were described by comparing their sensitive pathways and specific genes with those of other neurons. A blueprint of C3:Ex.Neuron was constructed based on the differentially expressed genes, such as TFs and long non-coding RNAs (lncRNAs), and the TF-target regulatory network according to the activity changes in AD compared to WT 1.5, 4, 4.7, 7, and 13 months. C3:Ex.Neuron was divided into four subtypes (C3.1–4) based on the differentially expressed genes. C3.1 neurons exhibited high expression of netrin G1 (*Ntng1*) and similar patterns of abundance changes and enriched functional pathways as did C3:Ex.Neuron.

## Materials and Methods

### Data Resources

The single-nucleus RNA raw data used in this study were obtained from the study conducted by Habib et al. (2020) (GEO: GSE143758).

### Sequence Alignment and Generation of Gene-Cell Matrices

The Cellranger toolkit (v.6.0.0)^1^ was used to process the raw-sequencing data *via* de-multiplexing. Sequences were aligned to the mouse reference genome (GRCm38) using Spliced Transcripts Alignment to a Reference (STAR) (Dobin et al., 2013) and barcode processing to generate gene-cell matrices used for downstream analyses.

### Single-Cell RNA Sequencing Data Filtering and Normalization

Cells with <800 unique molecular identifiers (UMIs) containing <400 genes or >5,000 UMIs were discarded from downstream analyses, as these values implied that the cells were dead or multiplet, respectively. Only genes expressed in at least 10 cells were considered for further analysis, and mitochondrial genes were removed. The final dataset consisted of 49,546 cells and 19,934 genes. Normalized UMI counts for each cell were determined by dividing each UMI count by the total UMI count of the cells (counts per million, CPM), log transformed after the addition of 1 (log1pCPM), and scaled by the median CPM across all cells. Analysis was performed using Seurat 4.0.3 (Satija et al., 2015; Butler et al., 2018; Stuart and Satija, 2019; Hao et al., 2021).

### Batch Effect Removing, Cell Clustering, and Cell Type Identification

The top 2,000 highly variable genes (HVGs) were selected for each sample before integrating different batches of single-cell data. The batch effect of the data was removed by Seurat 4.0.3, and the HVGs were conserved. The principal component analysis (PCA) of the HVGs determined the ranking of principal component contributions, and the top 15 principal components were selected for downstream analysis (Supplementary Figure 1A). After batch effect removal (Supplementary Figure 1B), a resolution of 0.4 was selected to cluster the cells into 13 populations (Supplementary Figure 1C). This value was deemed appropriate, as the top 10 cell groups highly expressed genes were showed in heatmap (Supplementary Figure 2A). The cell types were classified based on previous reports (Mathys et al., 2019; Habib et al., 2020) and the CellMarker database (Zhang et al., 2019). The G-test was used to determine the change in the proportion of cell groups between AD and WT mice at different time points.

### Gene Set Variation Analysis, Gene Set Enrichment Analysis, and Differential Gene Expression Analysis

Two thousand cells contained all the identified cell types were randomly selected to explore the function of different cell types *via* GSVA analysis (Hanzelmann et al., 2013). Gene-cell matrices and cell-group files were generated, and GSEA analysis (Mootha et al., 2003; Subramanian et al., 2005) was performed to identify changes in functional pathways between groups. Differentially expressed genes were analyzed using Seurat 4.3.0 (Satija et al., 2015; Butler et al., 2018; Stuart and Satija, 2019; Hao et al., 2021).

### Single-Cell Regulatory Network Inference and Clustering Analysis

The single-cell regulatory network inference and clustering (SCENIC) analysis (Aibar et al., 2017; Van de Sande et al., 2020) was used to reflect the state of cells and construct a gene regulatory network with TF. First, a co-expression module of TF and its potential target genes were constructed. Second, cis-regulatory motifs were used to remove indirectly regulated genes from these modules; TFs and their target genes constituted regulons (Aibar et al., 2017; Van de Sande et al., 2020). Finally, the activity of regulons was quantified by enriching the target genes of these TFs.

## Results

### Re-construction of the Hippocampal Cell Atlas in Alzheimer’s Disease and Wild-Type Mice

**FIGURE 1:**
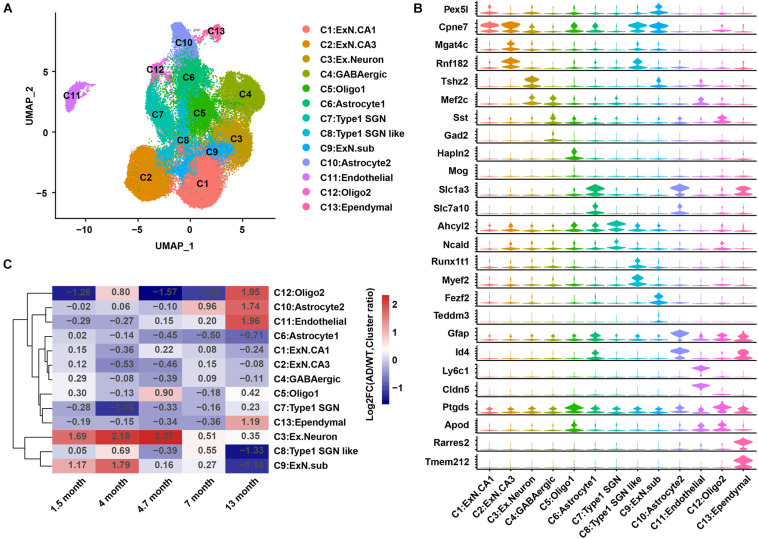
A cell atlas of hippocampal cells in 5XFAD (AD) mice and WT mice. **(A)** Cell map of mouse hippocampus. Each dot represents a cell and is colored according to a cluster. **(B)** Violin plots showing the distribution of the expression of specific marker genes in each cell group. **(C)** Heatmap showing the changes in relative abundance of the 13 cell groups in AD mice compared to that in WT mice at 1.5, 4, 4.7, 7, and 13 months. AD, Alzheimer’s disease; WT, wild-type.

**FIGURE 2:**
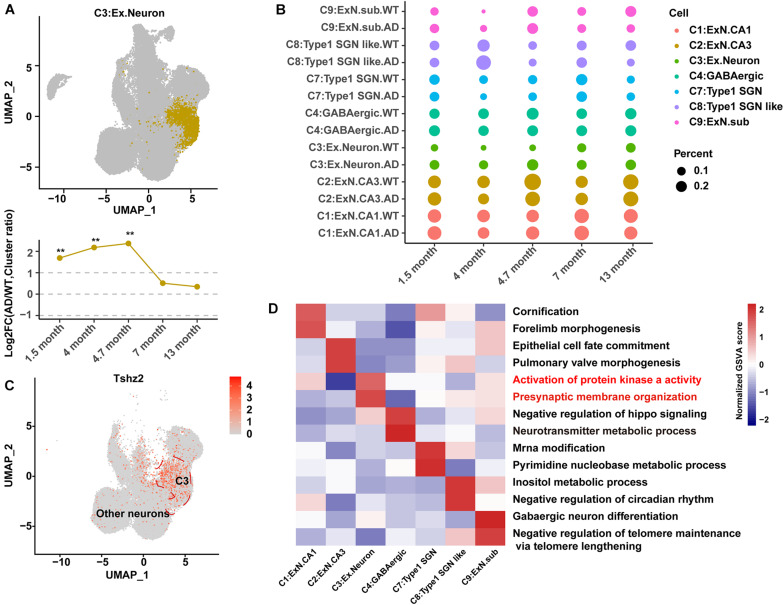
Identification and characteristics of C3:Ex.Neuron as an AD-associated neuronal population. **(A)** Uniform Manifold Approximation and Projection (UMAP) plot showing the distribution of excitatory neurons in the hippocampus. The bottom graph shows the ratio changes in AD excitatory neurons compared to those in WT neurons at 1.5, 4, 4.7, 7, and 13 months. G-test, **p < 0.001. **(B)** Dot plot showing the ratio of neuron cell groups between AD and WT mice. The dot size represents the percentage of cells. **(C)** UMAP plot showing the expression of specific genes in excitatory neurons compared to that in other neuron cell groups of the hippocampus. **(D)** Heatmap showing the gene set variation analysis (GSVA) scores to reveal the sensitive functional pathway of different cell groups. The red color indicates that the pathway is associated with AD. Statistical *p*-values < 0.01, log2FC > 1, or log2FC <–1, determined using the G-test, were considered to be significant. AD, Alzheimer’s disease; WT, wild-type.

An integrated analysis of the single-cell data from GEO: GSE143758 (Habib et al., 2020) was performed to re-construct the hippocampal cell atlas in AD and WT mice. These 13 cell populations were designated as excitatory neurons CA1 (C1:ExN.CA1); excitatory neurons CA3 (C2:ExN.CA3); C3:Ex.Neuron; gamma-aminobutyric acid neuron (C4:GABAergic); oligodendrocytes 1 (C5:Oligo1); C6:Astrocyte1; Type 1 spiral ganglion neurons (C7:Type1 SGN); Type 1 spiral ganglion neurons like (C8:Type1 SGN like); excitatory neurons subiculum (C9:Ex.N.sub); C10:Astrocyte2; C11:Endothelial; oligodendrocytes 2 (C12:Oligo2); and C13:Ependymal, based on previous studies (Mathys et al., 2019; Habib et al., 2020) and the CellMarker database (Zhang et al., 2019; Figure 1A). Two representative genes for each cell population were showed in a violin chart (Figure 1B), and the expression distribution of classic marker genes was shown by the Uniform Manifold Approximation and Projection (UMAP) diagram (Figure 2C and Supplementary Figure 2B).

The cell number in each sample and the proportion of each cell group among the samples were calculated. Samples at 14 and 10 months were removed because of their extremely low cell numbers or abnormal cell ratios (Supplementary Figures 3A,B). The differences in cell ratios between the WT and AD mice analyzed at the five qualified time points (1.5, 4, 4.7, 7, and 13 months) showed that AD pathology was under coordinated control, and the ratios for most cell groups were changed during AD development (Figure 1C). The relative abundance (cell ratio in AD/cell ratio in WT) of two cell clusters (C3:Ex.neuron and C10:Astrocyte2) was significantly changed (Figure 2A and Supplementary Figure 4A). C10:Astrocyte2 was stable in AD mice from 1.5 to 4.7 months, but increased at 7 and 13 months (Supplementary Figure 4A). The changes in C10:Astrocyte2 abundance, together with its enriched functional pathways in immune-related terms (Supplementary Figures 4B–D), were consistent with previous reports (Ahmad et al., 2019; Habib et al., 2020).

### Identification and Characterization of a Group of Alzheimer’s Disease-Associated C3:Ex.Neuron

The relative abundance of C3:Ex.Neuron to the total populations dramatically increased in AD mice compared with that in WT mice at the earliest collected stage (1.5 months) and 4 months (Figures 2A,B). The biomarker for C3:Ex.Neuron was identified as teashirt zinc finger homeobox 2 (TSHZ2), a protein previously identified in excitatory neurons (Figure 2C; Habib et al., 2020). The characteristics of C3:Ex.Neuron and the other identified neurons were determined *via* GSVA analysis. C3:Ex.Neurons were highly enriched for the AD-associated terms “ACTIVATION OF PROTEIN KINASE A ACTIVITY” and “PRESYNAPTIC MEMBRANE ORGANIZATION” (Figure 2D; Zhang et al., 2006). Although several other neurons showed enrichment of AD-associated terms, the relative abundance changes of the other types of neurons were not significant (Figure 2B).

**FIGURE 3:**
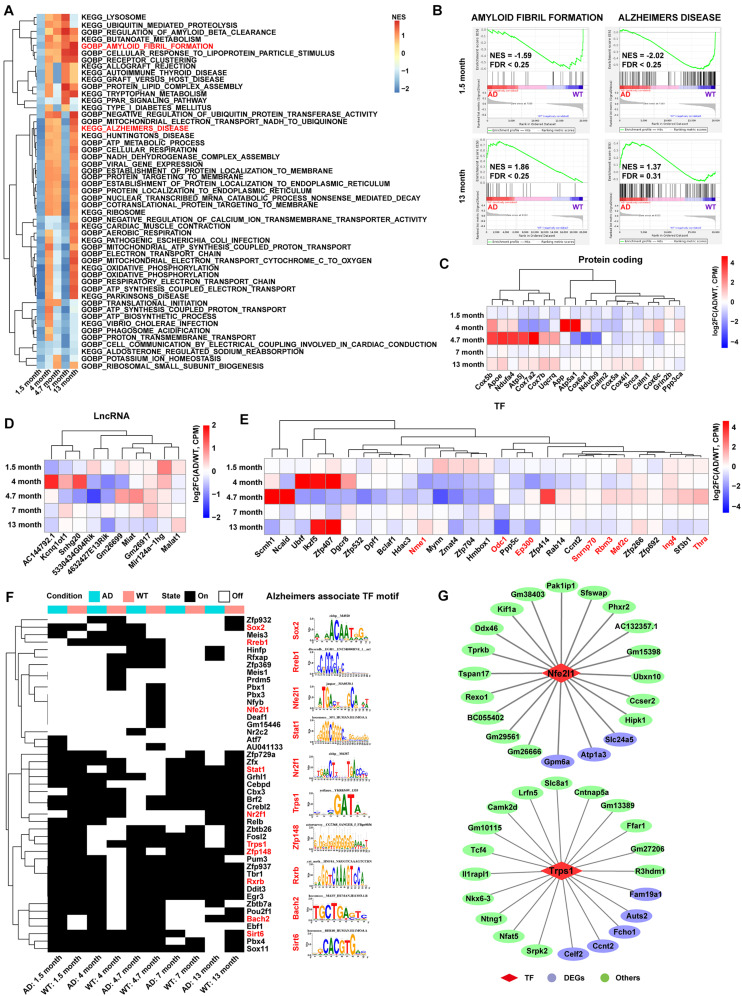
Functional pathway and gene landscape analysis for C3:Ex.Neuron. **(A)** Heatmap displaying the significantly altered pathways of AD compared with WT excitatory neurons at 1.5, 4, 4.7, 7, and 13 months, as assessed *via* gene set enrichment analysis (GSEA). The pathways with a false discovery rate (FDR) < 0.25 and normalized enrichment score (NES) > 1 were considered to be significantly enriched. **(B)** GSEA showing the excitatory neurons involved in AD-related pathways at 1.5 and 13 months. **(C–E)** Heatmaps showing differential expression of protein-coding genes, lncRNAs, and TFs in AD compared with that in WT mice at different time points in excitatory neurons. Red-colored genes represent Alzheimer’s disease recorded in the molecular signatures (Msigdb) database. **(F)** SCENIC analysis revealing the switch of TF activity between AD and WT mice in excitatory neurons. The TF motifs with an activity difference in at least one time point were presented. Red-colored TFs represent Alzheimer’s disease recorded in the molecular signatures (Msigdb) database. **(G)** The network showing the TFs and their target genes in excitatory neurons. AD, Alzheimer’s disease; WT, wild-type; SCENIC, Single-cell regulatory network inference and clustering.

The C3:Ex.Neuron expression matrix in both AD and WT mice was constructed to explore the main pathway changes in C3:Ex.Neuron *via* GSEA analysis with reference to Gene Ontology (GO) and Kyoto Encyclopedia of Genes and Genomes (KEGG) annotation. Pathways significantly enriched in at least two time points are highlighted in Figure 3A. Along with the time extension, the changes of signal pathways showed a dramatic difference between AD and WT mice. It is worth mentioning that the pathways of AMYLOID FIBRIL FORMATION and ALZHEIMERS DISEASE were downregulated in C3:Ex.neuron at the 1.5 months of AD mice (Figures 3A,B). Together, our analyses indicated that C3: Ex.Neurons are a specific group of neurons that are associated with AD.

### Mis-Regulation of Long Non-Coding (lnc)RNA, Transcriptional Factors, and Transcriptional Factor-Target Regulatory Networks in C3:Ex.Neuron

Analysis of C3:Ex.Neuron identified a different expression gene (DEG) pool with 415 genes at 1.5, 4, 4.7, 7, and 13 months. The genes with *p*-value (Wilcoxon rank sum test) <0.05 and log2FC >0.25 were considered to be upregulated, and genes with *p*-value (Wilcoxon rank sum test) <0.05 and log2FC <−0.25 were considered to be downregulated (Supplementary Figure 5A and Supplementary Table 2). A second gene pool with 161 genes related to AD in C3:Ex.Neurons was constructed following GO and KEGG analyses (Supplementary Table 3). The two gene pools shared 19 significantly regulated genes, as assessed *via* hypergeometric tests (Supplementary Figure 5B). This included apolipoprotein E (*Aope*), with particular alleles of this gene correlated with a predisposition for developing AD (Riedel et al., 2016; Serrano-Pozo et al., 2021). Interestingly, most of these 19 overlapping genes were downregulated at the early stages (1.5 months) in AD mice (Figure 3C). lncRNAs play important roles in AD. For example, *BDNF-AS* was reported to negatively regulate neurotrophic factor expression and promote the pathogenesis of AD (Guo et al., 2018), and *LoNA* was reported to be involved in the regulation of memory in *APP/PS1* transgenic mice (Li et al., 2018). Our analysis revealed 10 differentially expressed lncRNAs, such as five downregulated lncRNAs (*AC144792.1*, *Kcnq1ot1*, *Snhg20*, *5330434G04Rik*, and *4632427E13Rik*) and five upregulated lncRNAs (*Gm26699*, *Miat*, *Gm26917*, *Mir124a-1hg*, and *Malat1*; Figure 3D).

Transcription factors are driving factors in mediating the transition of AD (Grubman et al., 2019). Our results identified the differentially expressed TFs in C3:Ex.Neuron that were associated with AD, through the Msigdb database (Figure 3E). Regulons (transcription factors and their target genes) were constructed to assess TFs’ activities *via* SCENIC analysis and TFs with the activity difference at least one time point were displayed (Figure 3F). A full list of identified TFs and their targets is shown in Supplementary Table 4. Two representative TFs with their top 20 targets are shown in Figure 3G.

Single-nucleus sequencing from the prefrontal cortex of 48 individuals with varying degrees of AD pathology had been published (Mathys et al., 2019). In this report, the authors divided cells into six main groups, excitatory neurons (Ex.Neuron), inhibitory neurons (In.Neuron), astrocytes, oligodendrocytes (Oligo), oligodendrocyte precursor cells (OPC), and microglia. To test whether the DEGs identified from AD mice could be observed from the patients with AD, we downloaded the expression data from their Supplementary Table 2 (Mathys et al., 2019, Nature) and focused on the data of no-pathology (Healthy) to early-pathology (AD-early). We found that the homologous genes of makers in our C3:Ex.Neuron, *TSHZ2*, and *MEF2C*, were also highly expressed in the human Ex.Neurons from both Healthy and AD-early groups (Supplementary Figure 6A), suggesting the similarity of the mouse C3:Ex.Neuron and human Ex.Neuron. Recall that we have identified 117 DEGs that were either upregulated or downregulated in the C3:Ex.Neuron of 1.5 months AD mice, compared with the same aged WT mice (Supplementary Figure 5A). In the work from Mathys et al. (Mathys et al., 2019), the authors have used two models to identify the regulated DEGs: Ind.model and Ind.Max.model. The authors found 4,928 DEGs by Ind.model and 1,151 DEGs by Ind.Max.model. We applied our identified mouse DEGs to the AD patient dataset and found 104 homologous genes (88.9% to our identified 117 mouse DEGs) between the two datasets. Among these 104 homologous genes, we found that 48 and 29.8% exhibited similar upregulated or downregulated changes in the AD-early group by comparing with the Ind.model and Ind.Max.model, respectively (Supplementary Figure 6B).

In summary, our results showed that both mouse C3:Ex.Neuron and Ex.Neuron from AD-early patients share the similarity in their dysregulated gene expression profiles. Our identified DEG blueprint of excitatory neurons will provide insights into the pathogenic regulatory mechanism of AD progression.

### Subtype Analysis of C3:Ex.Neuron Revealed Similarity Between C3.1 and C3:Ex.Neuron

**FIGURE 4:**
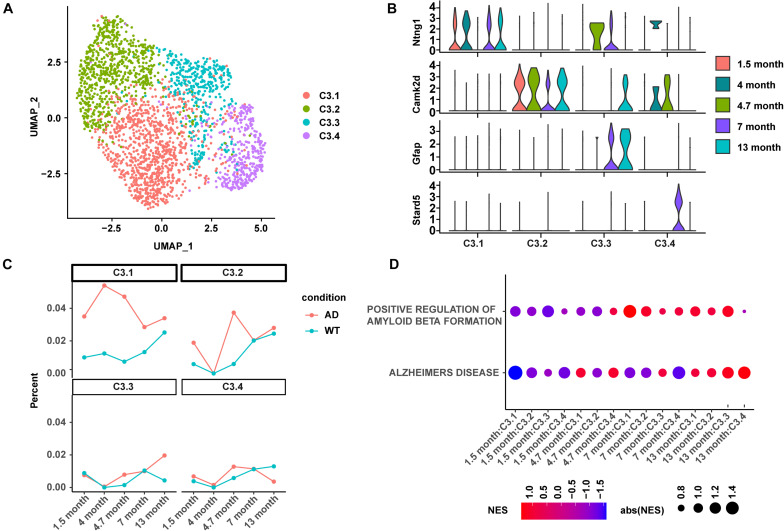
Subtype analysis of C3:Ex.Neuron revealed similarity between C3.1 and C3:Ex.Neuron. **(A)** UMAP plot showing four subtypes of C3:Ex.Neuron. Each dot represents a cell colored according to each cluster. **(B)** Violin plots showing the distribution of the subtype-specific markers of C3:Ex.Neuron. **(C)** The ratio of four excitatory neuron subtypes between AD and WT mice at 1.5, 4, 4.7, 7, and 13 months. **(D)** GSEA analyses reveal the enriched AD directly associated terms in applicable subtypes of C3:Ex.Neuron at 1.5, 4.7, 7, and 13 months of AD and WT mice. UMAP, Uniform Manifold Approximation and Projection; GESA, Gene set enrichment analysis; AD, Alzheimer’s disease; WT, wild-type.

Four C3:Ex.Neuron subtypes were identified and named as C3.1, C3.2, C3.3, and C3.4 using mutual nearest neighbors (MNN; Figure 4A). The abundance of differentially expressed genes was calculated using a negative binomial test and controlled false discovery rates (FDRs) following the Benjamini–Hochberg procedure. C3.1, C3.2, C3.3, and C3.4 were characterized by the biomarkers *Ntng1*, *Camk2d*, *Gfap*, and *Stard5*, respectively (Figure 4B). The analyses of the subtype proportion showed distinct changing patterns among the four identified subtypes during the development of AD (Figure 4C). Overall, the proportion of C3.1 showed to be upregulated in AD mice and exhibited a similar abundance pattern to that of C3:Ex.Neuron: increased from 1.5 to 4.7 months and then gradually dropped (Figure 4C). Great vibration with a circadian rhythm-like pattern was observed for C3.2 (Figure 4C). Both C3.3 and C3.4 started with relatively unchanged levels as the WT mice at 1.5 months and showed a slight increase at 4.7 months, but ended up with an increased level of C3.3 and a decreased level of C3.4 at 13 months in AD mice compared with WT mice. Consistently with its similarity to the C3:Ex.Neuron in abundance, the GSEA analysis showed that C3.1 was tightly associated with the downregulated AD-related terms “ALZHEIMERS DISEASE” and “POSITIVE REGULATION OF AMYLOID BETA FORMATION” at the very early stage in AD mice (1.5 months; Figure 4D). Taken together, our results revealed four subtypes of C3:Ex.Neuron and showed that only the subtype C3.1 exhibited similar characteristics of C3:Ex.Neuron.

## Discussion

The study on the pathogenic mechanism of AD is of great significance for its treatment, and single-cell sequencing-based analysis will provide novel insights of how the individual cell populations contribute to AD and the previously unidentified cellular mechanisms. In the present study, we identified a group of C3:Ex.Neuron being upregulated in abundance during the very early stages of the AD mouse model. The distinct regulatory patterns of its four subtypes of neurons (C3.1–4) in abundance and functional pathways suggest a cell-subtype-specific contribution to the disease escalation and coordinated control of AD through multiple cell populations. In addition, our observations of the downregulated AD-associated pathways in C3:Ex.Neuron and one of its subtypes (C3.1) suggested that C3:Ex.Neuron may play an anti-AD role through its subtype C3.1 which is highly expressed with Ntng1.

Netrin G1 is an anchored protein, located in cortex layer V neurons, Ntng1 binds to Netrin G ligand (NGL1) to mediate subcerebral projection. The previous report showed that knockdown of Ntng1 led to reduced accumulation of microglia along axons and increased neuronal loss (Fujita et al., 2020). In addition, mutation of Ntng1 has been suggested as a causative factor for Rett syndrome-like disorders in patients, such as intellectual disability, epilepsy, and autism (Ricciardi et al., 2012). Currently, the role of Ntng1 in AD has not been explored. By analyzing the publicly available database, we found that the expression level of Ntng1 showed a downregulated trend in hippocampus of patients with AD than the normal individuals (Supplementary Figure 7; Xu et al., 2018). We hypothesize that the increased abundance of Ntng1 highly expressed C3.1 excitatory neurons is a rapid response of the hippocampus to protect neurons from neurodegenerative damages at the onset stage of AD or under other neurodegenerative initiation stages because the positive terms of AD progression are downregulated in C3.1 excitatory neurons at the very early stage in AD mice. We further hypothesize that in the case of the 5XFAD mouse model or the patients with AD, the driving factors of AD are robust enough to overcome such increase and then trigger the drop of C3.1 neurons abundance. In the present study, we start to observe the reduction trend of C3.1 excitatory neurons at 4.7 months in AD mice after its peak at 4 months. The drop of C3.1 from its peak may represent an un-reversible transition of AD. Supporting our hypothesis and, interestingly, the drop of C3.1 neurons in abundance correlates with the increase of C10:Astrocyte2, an astrocyte population that has been found to emerge or increased in normal aging mice and humans (Figure 2A and Supplementary Figure 4A; Habib et al., 2020).

To date, the studies on AD through single-cell sequencing are mainly focused on the exploration of glial cells, such as microglia (Keren-Shaul et al., 2017; Olah et al., 2020) and astrocytes (Habib et al., 2020). Based on the same mouse model system (5XFAD mouse), our work found a subset of excitatory neurons associated with AD. Interestingly, we found that the DEGs identified from these mouse excitatory neurons share a high level of similarity with the excitatory neurons defined from patients with AD at the early stages.

In summary, by characterizing the features of C3:Ex.Neuron and its subgroup cells in AD mice, we identified a novel subset of excitatory neurons that may develop into novel strategies for AD prevention.

## Data Availability Statement

The original contributions presented in the study are included in the article/Supplementary Material, further inquiries can be directed to the corresponding author.

## Author Contributions

XW and FS initiated the project and designed the analytical process. XW organized and supervised the whole project. FS, MW, and QG performed all the bioinformatic analysis. BZ provided critical input for the project design and results analyses. FS drafted the manuscript. XW and FS finished the manuscript with input from MW, QG, and BZ. All authors contributed to the article and approved the submitted version.

## Conflict of Interest

The authors declare that the research was conducted in the absence of any commercial or financial relationships that could be construed as a potential conflict of interest.

## Publisher’s Note

All claims expressed in this article are solely those of the authors and do not necessarily represent those of their affiliated organizations, or those of the publisher, the editors and the reviewers. Any product that may be evaluated in this article, or claim that may be made by its manufacturer, is not guaranteed or endorsed by the publisher.

## Footnotes

1
https://support.10xgenomics.com
