# Supplementary material for: Characterization of Alzheimer’s Disease-Associated Excitatory Neurons via Single-Cell RNA Sequencing Analysis
Source: Front Aging Neurosci. 2021 Nov 8;13:742176. doi: 10.3389/fnagi.2021.742176 (PMC8606650; doi:10.3389/fnagi.2021.742176)
Supplement: Supplementary file 1 [file Data_Sheet_1.pdf]

frontiers

Supplementary Material

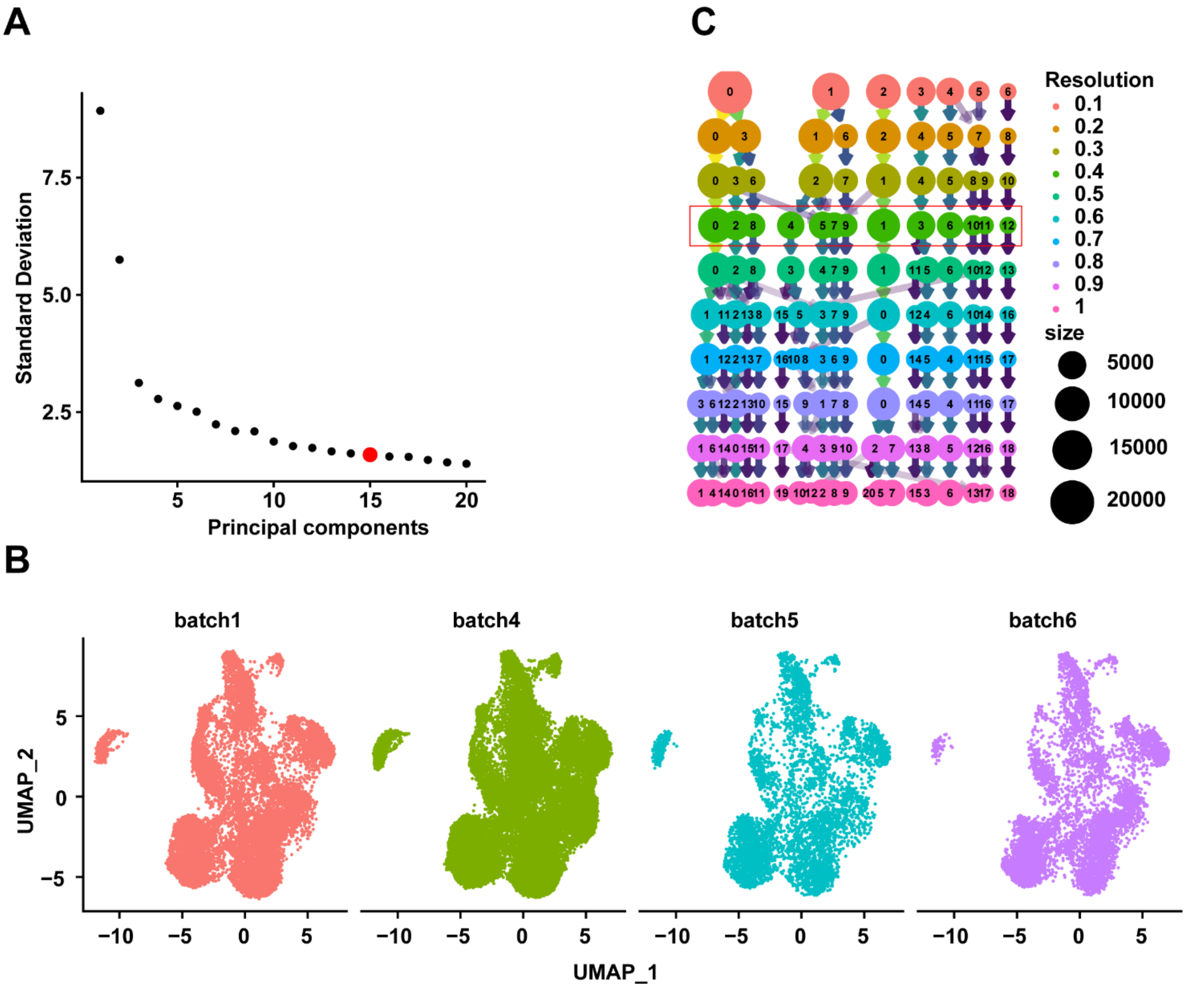

Supplementary Figure S1. Parameter selection and batch effect removal.

(A) Scree plot shows the contribution of different principal components.

(B) A cell atlas of hippocampal cells in different batches. Each dot represents a cell; colored by batch.

(C) Number of cell clusters with indicated resolutions.

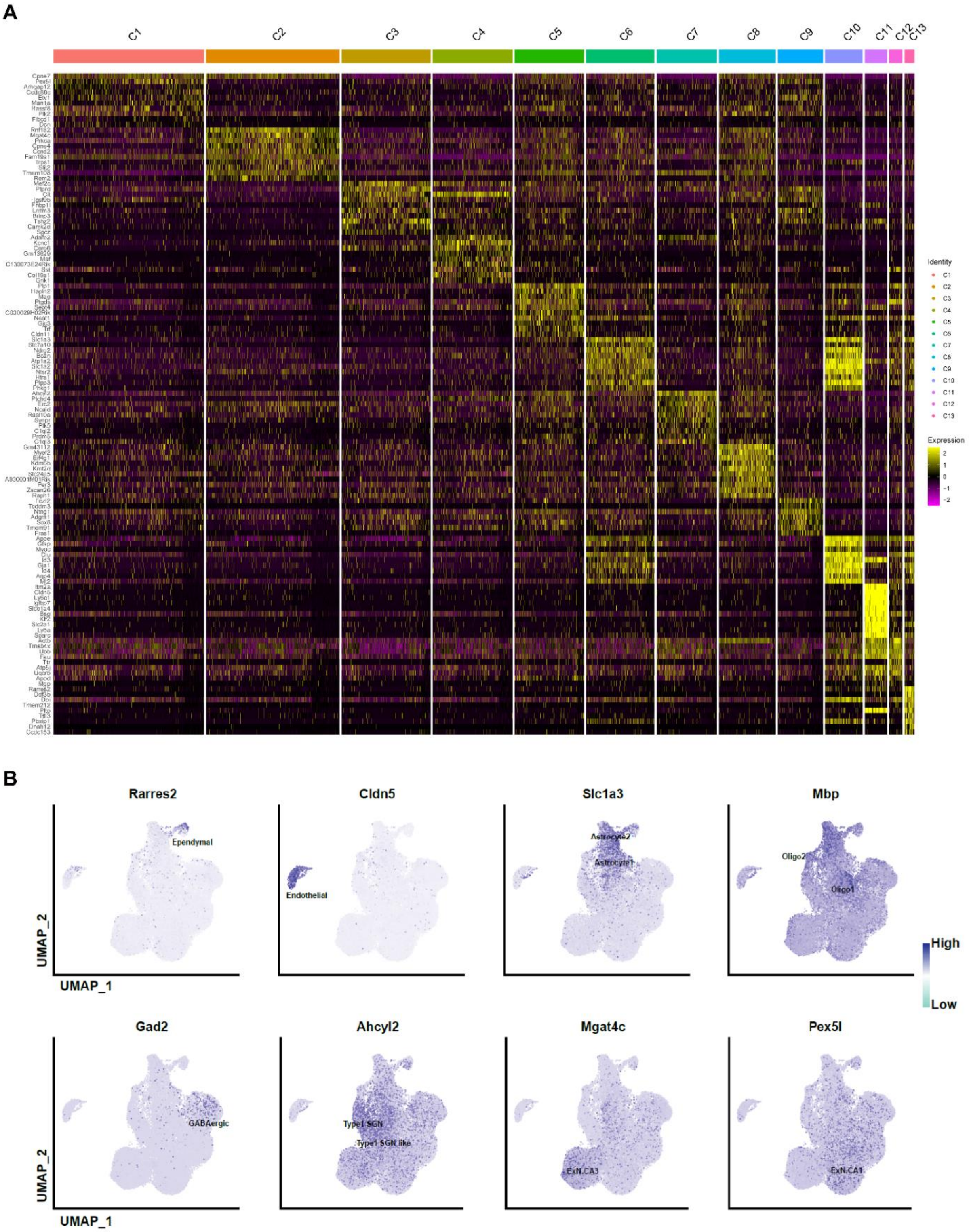

Supplementary Figure S2. Cell cluster specific expression genes.

(A) Heatmap shows top 10 genes of each cluster.

(B) UMAP plot shows the expression distribution of C13:Ependymal, C11:Endothelial, C6:Astrocyte1 and C10:Astrocyte2, C5:Oligo1 and C12:Oligo2, C4:GABAergic, C7:Type1 SGN and C8:Type1 SGN like, C2:ExN.CA3, and C1:ExN.CA1.

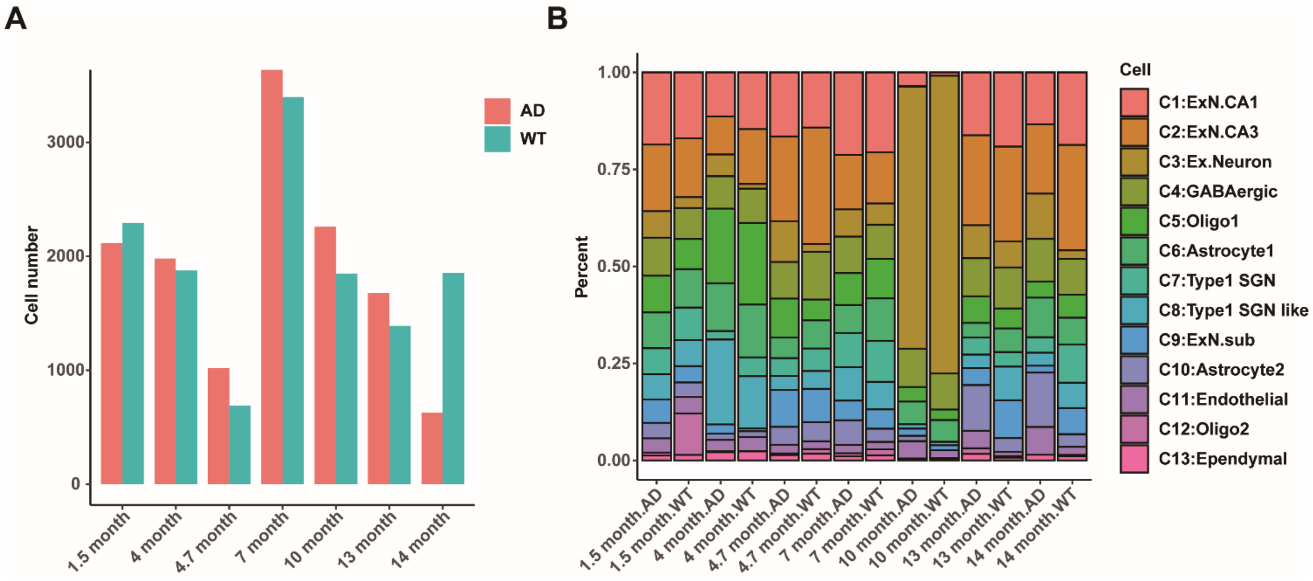

Supplementary Figure S3. Cell number and cell cluster ratio in different samples.

(A) Histogram shows cell number in different samples. (B) Stacked bar chart shows cell cluster ratio in different samples.

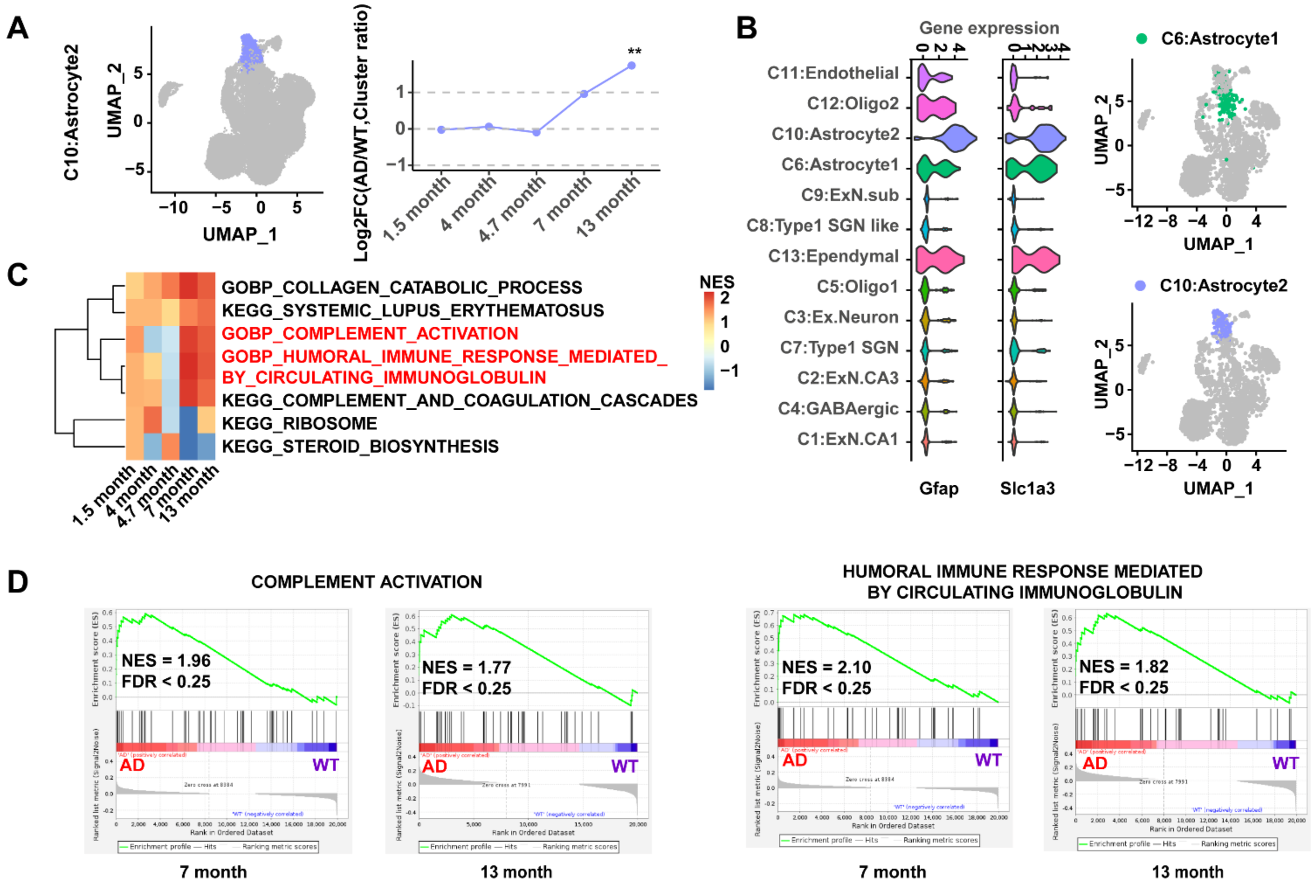

Supplementary Figure S4. Identification of C10:Astrocyte2 as immune-associated non-neural population and its characteristics.

(A) UMAP plot shows distribution of C10:Astrocyte2 in hippocampus. The below graph shows the changes of ratio of C10:Astrocyte2 compared AD to WT at 1.5, 4, 4.7, 7, and 13 months. G test, **: p-value < 0.001. (B) Violin plot shows expression of C10:Astrocyte2 specific gene compared to other cell groups of hippocampus. (C) Heatmap displays the significant changed pathways compared AD to WT of C10:Astrocyte2 at 1.5, 4, 4.7, 7, and 13 months by GSEA analysis. (D) GSEA plot shows C10:Astrocyte2 immune-related pathways in 7 months and 13 months.

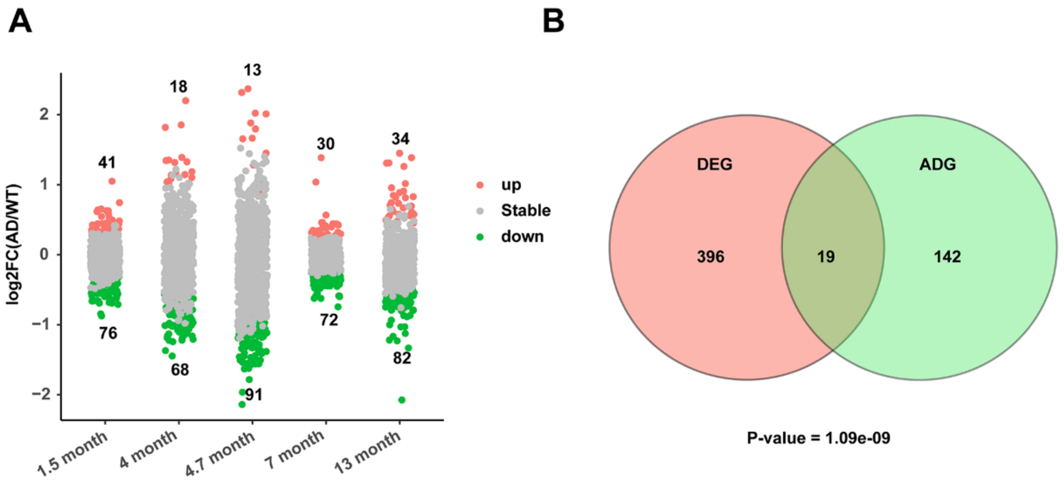

Supplementary Figure S5. Different expression genes of C3:Ex.Neuron between AD and WT.

(A) The scatter plot shows the number of different expression genes of C3:Ex.Neuron at different time points. (B) Venn plot shows the overlapping genes between different expression genes (DEG) of C3:Ex.Neuron and Alzheimer's disease genes(ADG), hypergeometric distribution test, p-value <0.01.

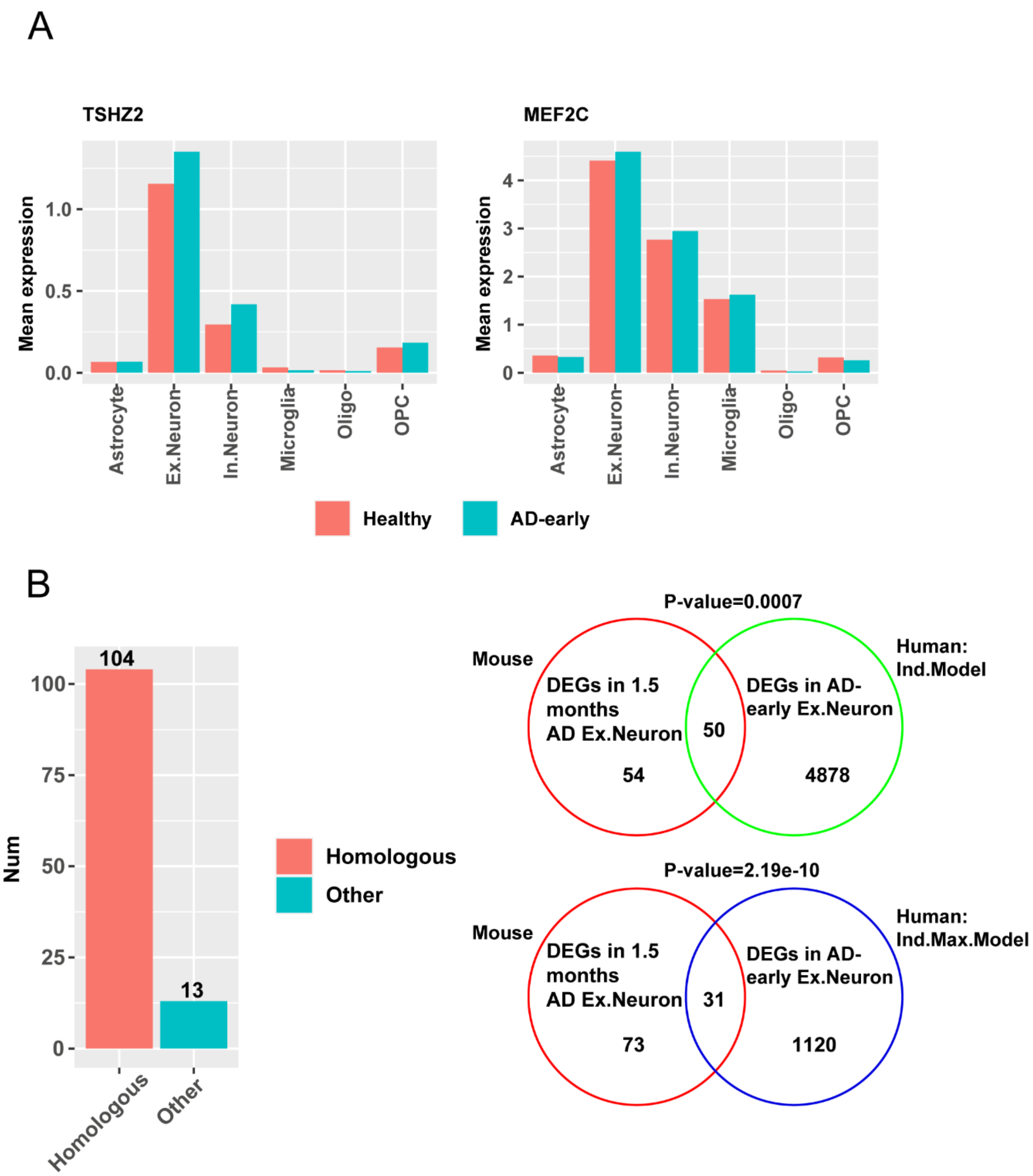

Supplementary Figure S6. Validation of Ex.Neuron and its different expression genes in AD-early human.

(A) Histogram analysis shows the high expression levels of TSHZ2 and MEF2C in human excitatory neurons based on Hansruedi Mathys et al., 2019, Nature.

(B) Left: Histogram shows the statistics on the homologous genes of DEGs of 1.5 months C3:Ex.Neuron corresponded to human.Upper right and bottom right: Venn plots show the overlapping genes between DEGs identified from C3:Ex.Neuron in 1.5 months AD mouse (Supplementary Table S2) and DEGs from Ex.Neuron in AD-early human (Hansruedi Mathys et al., 2019, Nature) with Ind.model (upper right) and Ind.Max.model (bottom right), respectively. Hypergeometric distribution test, p-value <0.01.

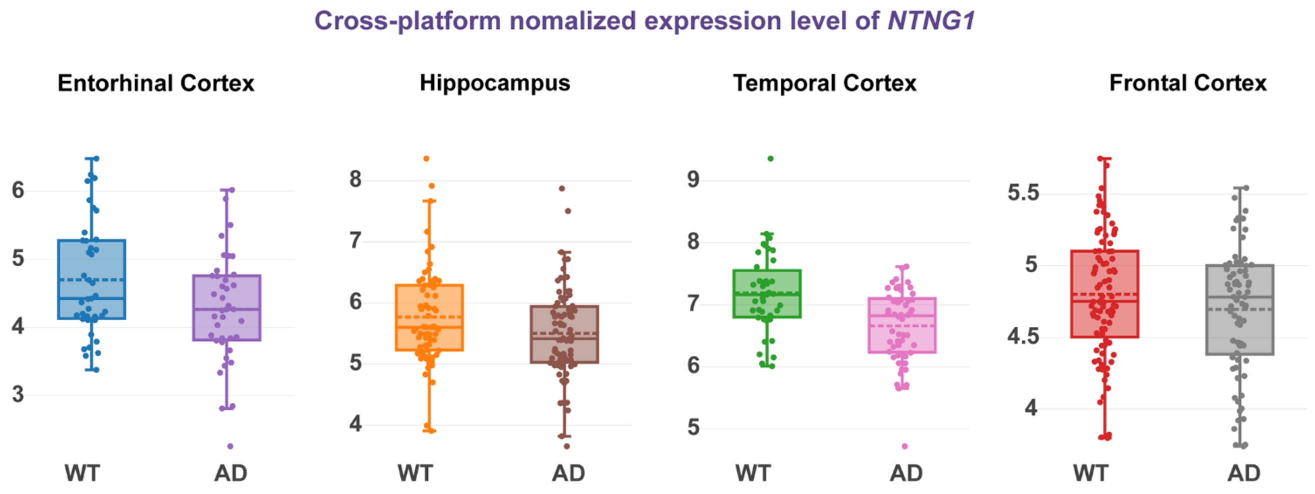

NTNG1: netrin G1		
Brain region	log2 FoldChange	P-value
Entorhinal Cortex	-0.36	0.048
Hippocampus	-0.26	0.058
Temporal Cortex	-0.55	0.000107804316648713
Frontal Cortex	-0.15	0.017

Supplementary Figure S7. Expression level of Ntng1 between AD and Control in human brain.
